# Severe falciparum malaria in pregnancy in Southeast Asia: a multi-centre retrospective cohort study

**DOI:** 10.1186/s12916-023-02991-8

**Published:** 2023-08-24

**Authors:** Makoto Saito, Aung Pyae Phyo, Cindy Chu, Stephane Proux, Marcus J. Rijken, Candy Beau, Htun Htun Win, Laypaw Archasuksan, Jacher Wiladphaingern, Nguyen H. Phu, Tran T. Hien, Nick P. Day, Arjen M. Dondorp, Nicholas J. White, François Nosten, Rose McGready

**Affiliations:** 1 Shoklo Malaria Research Unit, Mahidol-Oxford Tropical Medicine Research Unit, Faculty of Tropical Medicine, Mahidol University, Mae Sot, Thailand; 2 Centre for Tropical Medicine and Global Health, Nuffield Department of Medicine, University of Oxford, Oxford, UK; 3 Division of Infectious Diseases, Advanced Clinical Research Center, Institute of Medical Science, University of Tokyo, Tokyo, Japan; 4 Julius Global Health, Julius Center for Health Sciences and Primary Care, University Medical Center Utrecht, Utrecht University, Utrecht, The Netherlands; 5 Department of Obstetrics and Gynecology, University Medical Center Utrecht, Utrecht University, Utrecht, the Netherlands; 6 Oxford University Clinical Research Unit, Hospital for Tropical Diseases, Ho Chi Minh City, Vietnam; 7 Mahidol-Oxford Tropical Medicine Research Unit (MORU), Faculty of Tropical Medicine, Mahidol University, Bangkok, Thailand

**Keywords:** Severe malaria, *Plasmodium falciparum*, Pregnancy, Maternal mortality, Foetal loss, Small-for-gestational-age, Preterm birth

## Abstract

**Background:**

Severe malaria in pregnancy causes maternal mortality, morbidity, and adverse foetal outcomes. The factors contributing to adverse maternal and foetal outcomes are not well defined. We aimed to identify the factors predicting higher maternal mortality and to describe the foetal mortality and morbidity associated with severe falciparum malaria in pregnancy.

**Methods:**

A retrospective cohort study was conducted of severe falciparum malaria in pregnancy, as defined by the World Health Organization severe malaria criteria. The patients were managed prospectively by the Shoklo Malaria Research Unit (SMRU) on the Thailand-Myanmar border or were included in hospital-based clinical trials in six Southeast Asian countries. Fixed-effects multivariable penalised logistic regression was used for analysing maternal mortality.

**Results:**

We included 213 (123 SMRU and 90 hospital-based) episodes of severe falciparum malaria in pregnancy managed between 1980 and 2020. The mean maternal age was 25.7 (SD 6.8) years, and the mean gestational age was 25.6 (SD 8.9) weeks. The overall maternal mortality was 12.2% (26/213). Coma (adjusted odds ratio [aOR], 7.18, 95% CI 2.01–25.57, *p* = 0.0002), hypotension (aOR 11.21, 95%CI 1.27–98.92, *p* = 0.03) and respiratory failure (aOR 4.98, 95%CI 1.13–22.01, *p* = 0.03) were associated with maternal mortality. Pregnant women with one or more of these three criteria had a mortality of 29.1% (25/86) (95%CI 19.5 to 38.7%) whereas there were no deaths in 88 pregnant women with hyperparasitaemia (> 10% parasitised erythrocytes) only or severe anaemia (haematocrit < 20%) only. In the SMRU prospective cohort, in which the pregnant women were followed up until delivery, the risks of foetal loss (23.3% by Kaplan–Meier estimator, 25/117) and small-for-gestational-age (38.3%, 23/60) after severe malaria were high. Maternal death, foetal loss and preterm birth occurred commonly within a week of diagnosis of severe malaria.

**Conclusions:**

Vital organ dysfunction in pregnant women with severe malaria was associated with a very high maternal and foetal mortality whereas severe anaemia or hyperparasitaemia alone were not associated with poor prognosis, which may explain the variation of reported mortality from severe malaria in pregnancy. Access to antenatal care must be promoted to reduce barriers to early diagnosis and treatment of both malaria and anaemia.

**Supplementary Information:**

The online version contains supplementary material available at 10.1186/s12916-023-02991-8.

## Background

*Plasmodium falciparum* infection remains the main cause of death from malaria worldwide [1–3]. Although other malaria species can occasionally cause severe malaria and contribute to mortality [3], untreated severe falciparum malaria is commonly fatal [2]. If treated promptly with intravenous artesunate, however, the mortality of severe malaria is reduced by one-third compared with the previously recommended quinine [3–5]. Since 2006, intravenous artesunate has been recommended by the World Health Organization (WHO) as the first-line treatment for all patients with severe malaria, including pregnant women in all trimesters [6]. In pregnancy, the risk of developing severe malaria is higher than in non-pregnant women in both high- and low-transmission areas [7] (2–3 times higher in low-transmission areas [8]), and the mortality of severe malaria is usually reported as higher in pregnancy [2, 9, 10]. However, the Southeast Asian Quinine Artesunate Malaria trial (SEAQUAMAT), a cornerstone multi-centre randomised controlled trial (RCT) on the treatment of severe malaria, reported that mortality was lower in pregnancy (10%, 5/49) than in non-pregnant adults (19%, 65/337) [4]. Other studies have reported that mortality from severe malaria was not significantly different between pregnant and non-pregnant groups [8, 11]. These differences could be explained by the different inclusion criteria, within the definition of severe malaria, of sub-groups with a relatively good prognosis.

In contrast to the large prospective series of severe malaria in children and non-pregnant adults that have been reported [4, 12], there are a few studies on severe malaria in pregnancy [13]. Certain clinical or laboratory findings (e.g. coma, acidosis) are associated with an increased risk of death within the broader definition of severe malaria [5, 9, 14] but this has not been studied specifically in pregnancy. Although the priority in treating severe malaria in pregnancy is to save the life of the mother, severe malaria carries a substantial risk of adverse foetal and neonatal outcomes: a study in Rwanda showed the risks of stillbirth, preterm birth and low birth weight were higher in mothers who had severe malaria compared with those who had uncomplicated malaria [15]. In lower-transmission areas, these risks of adverse outcomes from severe malaria are regarded as higher [11]. Few studies have described foetal outcomes following severe malaria in pregnancy particularly beyond the acute phase. For these reasons, we retrospectively reviewed the records of severe malaria in pregnancy managed by the Shoklo Malaria Research Unit (SMRU) on the Thailand-Myanmar border, where pregnant women are followed prospectively in antenatal clinics until delivery, together with the data collected in prospective severe malaria studies of hospitalised patients in six Southeast Asian countries conducted or coordinated by the Mahidol-Oxford Tropical Medicine Research Unit (MORU) and the Oxford University Clinical Research Unit (OUCRU). Our aim was to describe maternal and foetal mortality and morbidity associated with severe falciparum malaria in pregnancy (as defined by the WHO severe malaria criteria) and to identify the factors predicting higher maternal mortality.

## Methods

### Study design and data sources

The data of severe falciparum malaria in pregnancy in Southeast Asia were derived from two different series: the SMRU pregnancy cohort on the Thailand-Myanmar border and the MORU/OUCRU clinical studies of hospitalised patients in Bangladesh, India, Indonesia, Myanmar, Thailand and Vietnam. Clinical data from birth records of pregnant women without severe malaria (i.e. uncomplicated falciparum malaria) collected from 1989 to 2020 at SMRU was additionally extracted for comparison of maternal and birth outcomes. We describe the SMRU cohort in detail and provide birth outcomes as these women were followed up systematically until delivery. The MORU/OUCRU studies were of patients hospitalised with severe malaria and were included to the analyses of maternal mortality, but these studies did not follow surviving women to delivery.

### Inclusion criteria

We used the current WHO severe malaria criteria [6] to define severe falciparum malaria (Additional file 1: Table S1) with some modifications related to the availability of laboratory tests (see below). Within the WHO severe malaria criteria, severe anaemia was defined as a haemoglobin < 7 g/dL (or haematocrit < 20%) with a parasite count > 10,000 /µL, and hyperparasitaemia was defined as an asexual parasitaemia of ≥ 10%. Pregnant or postpartum (up to 42 days from delivery) [16] women with severe malaria, based on the WHO severe malaria criteria [6], with parasitological confirmation of falciparum malaria (either by microscopy or rapid diagnostic test) were included. Non-falciparum malaria (e.g. vivax malaria) or women without asexual parasitaemia of *P. falciparum* were excluded.

### Study setting: SMRU

SMRU provides primary health care and antenatal care (ANC) for refugees and migrants in clinics along the North-western border of Thailand, an area of low seasonal malaria transmission [17, 18]. Services have been provided free of charge for all pregnant women attending the clinics since 1986. In the refugee camps, more than 90% of women attended antenatal care [18]. Pregnant women were invited to follow up actively every 1–2 weeks at ANC clinics until delivery. At each visit, a smear from a finger-prick blood sample was screened by microscopy for malaria parasites. Haematocrit was measured every 2–4 weeks. All episodes of anaemia (haematocrit < 30%) were treated with haematinics (ferrous sulphate 200 mg twice daily and folate 5 mg daily). Women were encouraged to deliver in the SMRU inpatient facilities. The best estimate of gestational age (EGA) was applied, either from early pregnancy ultrasound dating (available from 2001), the Dubowitz examination for newborn assessment, or from an ultrasound standardised symphysis fundal height growth chart.

### Clinical management of malaria at SMRU

At SMRU, women with malaria parasitaemia were treated with effective antimalarial drugs (either with quinine-based or artemisinin-based treatment, which was changed over time as described in Additional file 2: Table S2) even when asymptomatic. Pregnant women with falciparum malaria were admitted for supervised treatment, and close clinical and parasitological assessment. Based on clinical severity, pregnant women with severe malaria were divided in three clinical management groups (Table 1): severe malaria with organ dysfunction (i.e. any WHO severe malaria criteria other than hyperparasitaemia or severe anaemia); severe malaria with hyperparasitaemia only; severe malaria with severe anaemia only. Parasite count, haematocrit, blood glucose and urine dipstick were assessed on site. Parasite count was assessed every 4–6 h until negative twice except for cases with severe anaemia only, for whom it was assessed daily. Malaria pigment (haemozoin) in neutrophils or monocytes on admission was assessed as presence or absence. Haematocrit was assessed at least every 24 h. Blood glucose was measured every 4 h if the patient was unconscious. Blood transfusion was considered if the haematocrit was less than 20% (haemoglobin 7 g/dL) in symptomatic women, or in case of hypovolemic shock.

**Table 1:** Antimalarial management of pregnant women who fulfilled the World Health Organization severe malaria criteria [6] in two series: community-based and hospital-based cohorts

**Cohort and diagnostic group**	**WHO severe malaria criteria **[6]	**Antimalarial management**
**SMRU pregnancy cohort (community-based)**		
a. Severe malaria with severe anaemia only	Severe anaemia: haematocrit < 20% or haemoglobin < 7 g/dL, with parasitaemia > 10,000 /μL	If no other WHO severe malaria criteria, oral treatment: quinine-based treatment (1989–2002) or artemisinin-based treatment (after 1994)^a^
b. Severe malaria with hyperparasitaemia only	Hyperparasitaemia > 10% infected red blood cells	If no other WHO severe malaria criteria, intravenous quinine-based treatment (1989–1993) or oral artemisinin-based treatment with a longer duration (after 1994)^a^ A rescue intravenous treatment (1.2 mg/kg artesunate) was given if parasitaemia was higher than 95-percentile of the usual parasite clearance rate [19]
c. Severe malaria with organ dysfunction	WHO severe criteria other than severe anaemia and hyperparasitaemia (Additional file 1)	Intravenous quinine (1989–1999), intramuscular artemether (1993–2000) or intravenous artesunate (after 2000)^a^
**MORU/OUCRU severe malaria cohorts (hospital-based)**	Any WHO severe malaria criteria (Additional file 1)	Parenteral quinine, artesunate or artemether

*MORU* Mahidol-Oxford Tropical Medicine Research Unit, *OUCRU* Oxford University Clinical Research Unit, *SMRU* Shoklo Malaria Research Unit, *WHO* World Health Organization

^a^Details of treatment regimens over time are described in Additional file 2

As in many low-resource settings, the biochemical criteria which contribute to the definition of severe malaria could not be assessed routinely: raised serum bilirubin (jaundice), renal failure (anuria) and pulmonary oedema (respiratory failure requiring supplemental oxygen) were diagnosed clinically. Metabolic acidosis, which is based on the laboratory results as defined in the WHO severe criteria, was therefore not available for the analyses.

### Hospital-based cohorts

The MORU/OUCRU patients were from prospective clinical studies of hospitalised patients conducted in Southeast Asia and included data from three RCTs on severe malaria as described previously [4, 20, 21]. Briefly, the SEAQUAMAT study compared intravenous artesunate and intravenous quinine in Bangladesh, India, Indonesia and Myanmar [4]; the AQ study compared intramuscular artemether and intramuscular quinine in Vietnam [20], and the AAV study compared intramuscular artesunate and intramuscular artemether also in Vietnam [21]. These trials included pregnant women in the second or third trimesters of pregnancy who fulfilled any of the WHO severe malaria criteria (Additional file 1: Table S1). The SEAQUAMAT study used a *Pf*HRP2-based rapid diagnostic test (Paracheck, Orchid Biosystems, Goa, India) for inclusion. The quantitative assessment of blood smear was conducted later. Haematology and biochemistry were tested on site. Last menstrual period was used for estimating gestational age in these studies.

### Outcomes

The primary outcome was case fatality of mother (maternal mortality), and secondary outcomes included foetal loss, preterm birth and birthweight of the foetus. Foetal loss, including death in utero, was categorised as miscarriage (< 28 weeks) or stillbirth (≥ 28 weeks), depending on EGA. Preterm birth was defined as live birth before 37 complete weeks (37 weeks + 0 days) [22]. Small-for-gestational-age (SGA) was defined as birthweight < 10% using the international standard [23]. Low birth weight was defined as < 2500 g [22]. Live-born singleton babies without congenital abnormality were included in the analyses of birthweight.

### Statistical analysis

Median with interquartile range (IQR) was used for describing continuous characteristics, and Wilcoxon’s rank sum test was used for comparing them. For describing outcomes, confidence intervals of proportions were calculated by the Wilson method. The number of missing information was excluded from the denominators of proportions. For those women who had more than one episode of severe malaria, only the final severe episode was used for assessing pregnancy outcomes.

To characterise the predicting factors for maternal death, the baseline characteristics and the WHO severe malaria criteria were assessed using the pooled dataset of the two series. All the available WHO severe criteria, calendar year, maternal age, maternal body weight, parity, EGA at malaria, parasite density and presence of gametocytaemia, schizontaemia, malaria pigment either in neutrophils or monocytes and fever were assessed. Firth’s penalised logistic regression was conducted with fixed-effects for each site to take account of the variability among the sites. For missingness, joint-modelling multilevel multiple imputation was conducted using *jomo* command in R [24] (Additional file 3). A multivariable model for predicting maternal mortality was built by backward elimination using *p* < 0.05 by Wald test as the cut-off [25]. Calendar year and treatment (quinine-based treatment or artemisinin-based treatment) were a priori confounders and were adjusted for in the multivariable analyses. Sensitivity analyses excluding cases with prostration or multiple convulsions only [2] were conducted.

Pregnancy outcomes were described using the SMRU cohort only, where patients were followed up until delivery. Kaplan–Meier survival estimate was used for describing foetal losses (miscarriages and stillbirths) after severe malaria. Preterm birth was assessed among those who had severe malaria before 37 complete weeks. The prevalence of adverse pregnancy outcomes was described for the three clinical management groups. Proportions of these outcomes among women with uncomplicated falciparum malaria in pregnancy at SMRU were shown for reference. As a sensitivity analysis, assessment of birthweight was also conducted only for those who were weighed within 3 days of the date of birth [26]. Stata MP 16.1 (Stata Corp, US) and R (R Foundation for Statistical Computing, Austria) were used.

### Meta-analysis

A meta-analysis on the mortality from severe malaria in pregnancy in Asia was conducted, including the current study data. A previous systematic review on malaria in pregnancy in Asia [11] was conducted in 2012, and another review on the treatment of severe malaria in pregnancy [13] was conducted in 2015. Therefore, we updated the review with PubMed on 5/August/2022 by searching for more recent articles on severe malaria in pregnancy published after 2011, using the following search terms without any limitations: ("severe malaria" OR "cerebral malaria" OR "complicated malaria") AND (pregnancy). Meta-analysis was conducted using DerSimonian and Laird’s random effects with Freeman-Tukey Double Arcsine Transformation. *I*^2^ was used for quantifying heterogeneity. We used *metaprop* command in Stata [27].

## Results

**Fig. 1:**
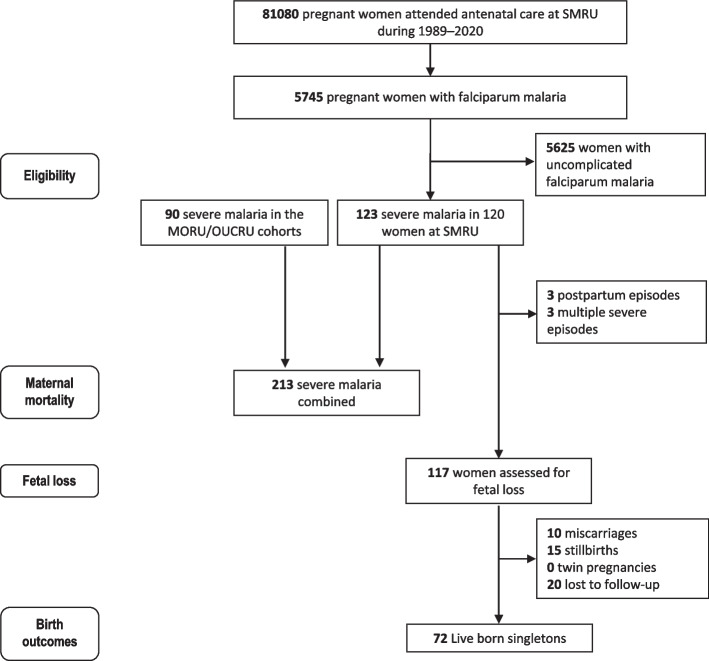
Flowchart of the pregnant women with severe malaria included in the analyses. MORU: Mahidol-Oxford Tropical Medicine Research Unit; OUCRU: Oxford University Clinical Research Unit; SMRU: Shoklo Malaria Research Unit

In total, 213 episodes of severe falciparum malaria in pregnancy were included in this pooled analysis (Fig. 1). These were in six Southeast Asian countries: Bangladesh (*n* = 18), India (*n* = 1), Indonesia (*n* = 10), Myanmar (*n* = 21), Thailand (*n* = 13) / Thailand-Myanmar border (*n* = 123) and Vietnam (*n* = 27).

### Baseline characteristics of the SMRU cohort

At SMRU between 1989 and 2020, there were 123 episodes in 120 pregnant women that fulfilled the WHO severe malaria criteria (Fig. 1). Overall, 14.6% (18/123) episodes were in the first trimester, 40.7% (50/123) were in the second trimester, 42.3% (52/123) were in the third trimester and 2.4% (3/123) were postpartum. During the latter part of this 31-year period, there was an active and highly successful programme of malaria elimination [17]: the last case of severe falciparum malaria in a pregnant woman was reported in 2013. More than half (61.8%, 76/123) of the severe episodes occurred in women who had not had any ANC visits before, or returned after > 14 days of absence from the weekly screening ANC programme. The three severe episodes in the postpartum period were in women who gave birth at home.

Hyperparasitaemia (52.6%, 61/116) and severe anaemia (42.9%, 48/112) were the most common severe malaria criteria fulfilled (Additional file 4: Table S3). The presenting haematocrit concentrations ranged from 9 to 43 (median 22.5, IQR 17.5–29)%. Cerebral malaria occurred in 19.0% (23/121).

Thirty-eight episodes were categorised as severe malaria with vital organ dysfunction, 50 episodes were categorised as severe malaria with hyperparasitaemia only, and 35 episodes were severe malaria with severe anaemia only (Table 1). The patients with vital organ dysfunction were slightly older (median 27 years, IQR 18–35), gestation was more advanced (median EGA 30.4 weeks, IQR 23.4–34.2) and higher proportions had schizontaemia (29.0%, 9/31) and gametocytaemia (45.2%, 14/31) in their admission blood smears than the other two groups (Table 2). The number of malaria episodes before the severe malaria episode was significantly larger in the severe anaemia only group (median 1, IQR 0–2) than the severe malaria with hyperparasitaemia only group (median 0, IQR 0–1, *p* = 0.02), but was not statistically different from the severe malaria with vital organ dysfunction group (median 0, IQR 0–1, *p* = 0.24).

**Table 2:** Demographic information for the Shoklo Malaria Research Unit (SMRU) severe malaria diagnostic groups and the Mahidol-Oxford Tropical Medicine Research Unit (MORU) / Oxford University Clinical Research Unit (OUCRU) hospitalised patients

Characteristic	SMRU			MORU/OUCRU
	Severe malaria with vital organ dysfunction (*n* = 38)	Severe malaria with hyperparasitaemia only (*n* = 50)	Severe malaria with severe anaemia only (*n* = 35)	Hospitalised severe malaria (*n* = 90)
Age (years)	27 [18–35]	23.5 [19–29]	25 [20–33]	25 [22–30] (*n* = 56)
Trimester				
First	11% (4/38)	18% (9/50)	14% (5/35)	9% (4/46)
Second	32% (12/38)	40% (20/50)	51% (18/35)	46% (21/46)
Third	50% (19/38)	42% (21/50)	34% (12/35)	46% (21/46)
Postpartum	8% (3/38)^a^	0	0	0
EGA (week)	30.4 [23.0–34.2]	25.4 [18.1–32.3]	23.9 [16.5–30.2]	26 [21.4–32] (*n* = 46)
Parity	2 [0–3]	1 [0–2]	1 [0–3]	No data
Gravidity	3 [1–5]	2 [1–3]	2 [1–4]	No data
Body weight (kg)	50 [45–54]	49 [44–52] (*n* = 49)	46 [42–53]	50 [44.5–54.5] (*n* = 88)
Fever > 37.5 °C on admission^b^	55% (16/29)	45% (21/47)	53% (18/34)	60% (44/73)
Days of fever	3 [2–5] (*n* = 29)	3 [2–4] (*n* = 48)	4 [1–5]	6 [4–8] (*n* = 49)
Species				
Pf mono-infection	91% (32/35)	92% (46/50)	89% (31/35)	No data
Pf + Pv coinfection	9% (3/35)	8% (4/50)	12% (4/35)	
Asexual parasitaemia load (/µL)	179,357 [112,538–372,655] (*n* = 31)	465,850 [409,958–531,288]	42,001 [16,052–64,433]	60,288 [13,188–31,6512] (*n* = 81)
Proportion of infected red blood cells (%)	5.4 [3.2–12.2] (*n* = 31)	12 [10.6–15.1]	2 [0.8–2.7]	2.1 [0.6–12.3] (*n* = 81)
Gametocytaemia	45% (14/31)	22% (11/50)	23% (8/35)	50% (7/14)
Schizontaemia	29% (9/31)	16% (8/50)	0% (0/35)	No data
Malaria pigment	87% (20/23)	79% (30/38)	33% (6/18)	89% (47/53)^c^
Haematocrit (%)	25 [19–29.5] (*n* = 28)	29 [25–33] (*n* = 48)	17 [16–18]	21 [17–26] (*n* = 73)
No previous ANC visits	32% (12/38)	28% (14/50)	37% (13/35)	No data
Days since last ANC^d^	14 [10–25] (*n* = 26)	13.5 [9–26.5] (*n* = 36)	18 [7–35] (*n* = 22)	No data
Treatment				
IV/IM artesunate	71% (24/34)	18% (9/50)	3% (1/35)	34% (31/90)
IV/IM artemether	13% (4/34)	0	0	17% (15/90)
IV/IM quinine	13% (4/34)	2% (1/50)	6% (2/35)	49% (44/90)
Oral ABT	0	58% (29/50)	66% (23/35)	0
Oral ABT + rescue IV	6% (2/34)	20% (10/50)	0	0
Oral quinine	0	2% (1/50)	26% (9/35)	0

*ABT* artemisinin-based therapy (either artesunate monotherapy, artesunate + clindamycin, or artemisinin-based combination therapy), *ANC* antenatal care, *EGA* estimated gestational age, *IM* intramuscular, *IV* intravenous, *Pf Plasmodium falciparum*, *Pv Plasmodium vivax*

Median [interquartile range] is shown. Severe anaemia is defined as a haemoglobin < 7 g/dL (or haematocrit < 20%) with a parasite count > 10,000/µL, and hyperparasitaemia is defined as more than asexual parasitaemia of > 10%. The number of patients assessed is shown in round brackets if not all were assessed. Two patients with both hyperparasitaemia and severe anaemia and another patient with hyperparasitaemia with hypoglycaemia on quinine were categorised into the uncomplicated hyperparasitaemia group

^a^Postpartum day 2, 2, 24

^b^Fever at any time was present in 29/32 in hyperparasitaemia only group, 32/35 in severe anaemia only group and 29/32 in severe malaria with organ dysfunction group

^c^Malaria pigment was assessed quantitatively in the MORU cohorts but was converted to presence or not in order to pool with data of the SMRU cohort

^d^Only women who started their ANC before severe malaria episode are included

First-line antimalarial treatment for severe malaria changed from quinine to artemisinin derivatives during the study period according to the local guidelines (Additional file 2: Table S2). All patients except two in the SMRU severe falciparum malaria with vital organ dysfunction category were treated parenterally: these two women had either jaundice or prostration at presentation but were not initially regarded as severe malaria, and so they were started on oral treatment. They then developed hyperparasitaemia and treatment was switched to parenteral artesunate. Women in the hyperparasitaemia only group were treated parenterally in 20.0% (10/50) of cases, and one dose of rescue parenteral treatment was given in a further 20.0% (10/50). In the severe anaemia only group, three patients (8.6%, 3/35) were treated parenterally. Blood transfusion was commonly required for women in the severe malaria with organ dysfunction group (55.9%, 19/34) and in the severe anaemia only group (86.7%, 26/30), but less often in the hyperparasitaemia only group (32.6%, 16/49).

### Maternal mortality in the SMRU cohort

At SMRU, there were 10 maternal deaths (10/123, 8.1%, 95% CI 4.5–14.3%). Mortality occurred only in women presenting with vital organ dysfunction; 26.3% (10/38) of this group died. There were no deaths in the other two groups (0/85) in which most women (72.9%, 62/85) were treated with oral antimalarials. Mortality was 8.3% (2/24) after intravenous artesunate, 25.0% (1/4) after intramuscular artemether, and 75.0% (3/4) after intravenous quinine. Most of the deaths (70.0%, 7/10) occurred within 24 h of admission. All deaths following intravenous artesunate or intramuscular artemether occurred within 24 h, while two patients died on day 4 of intravenous quinine. One woman died of severe malaria with eclampsia. Three other women had pre-eclampsia, and all survived.

### The hospital-based cohorts

The hospital-based cohorts included 90 hospitalised pregnant women who fulfilled the WHO severe malaria criteria between 1980 and 2016. The most common severe criterion was coma (52.2%, 47/90) (Additional file 4: Table S3). Only three women (3.4%, 3/89) had severe malaria with severe anaemia only. In these cohorts, 16 women died (16/90, 17.8%, 95% CI 11.2–26.9%). All women were treated parenterally. The maternal mortality was 16.1% (5/31) after parenteral artesunate, 13.3% (2/15) after parenteral artemether and 20.5% (9/44) after parenteral quinine.

### Mortality from severe malaria in Asia: meta-analysis

The maternal mortality of severe malaria in pregnancy reported in 14 studies [4, 10, 28–39] in the literature varied widely, ranging from 0 to 70.6% [4, 10, 28–39]. The overall pooled maternal mortality reported in studies of severe malaria in pregnancy in Asia including the current study was 25.1% (19 cohorts, range 0–70.6%, 95% CI 15.2–36.3%, *I*^2^ 87.1%). A forest plot is shown in Additional file 5: Figure S1.

### Factors associated with maternal death

The overall maternal mortality was 12.2% (26/213). Factors associated with maternal death were assessed by pooling the two series (Table 3). Although the point estimates of the odds ratio of mortality were higher than unity for all WHO severe malaria criteria, four clinical features were significantly associated with a higher risk of maternal death in univariable analyses: coma (22/70 vs 4/141, odds ratio [OR] 11.40, 95% CI 3.56–36.50, *p* < 0.0001), renal failure (5/17 vs 13/171, OR 4.79, 95%CI 1.21–18.99, *p* = 0.03), hypotension (2/6 vs 18/192, OR 10.83, 95%CI 1.84–63.89, *p* = 0.009) and respiratory failure (5/20 vs 16/171, OR 12.95, 95% CI 3.16–53.12, *p* = 0.0004). Neither EGA (continuous) nor trimester (categorical) was associated with maternal mortality. In the multivariable analysis adjusting for calendar year and treatment, coma (adjusted OR, 7.18, 95% CI 2.01–25.57, *p* = 0.002), hypotension (adjusted OR 11.21, 95%CI 1.27–98.92, *p* = 0.03) and respiratory failure (adjusted OR 4.98, 95%CI 1.13–22.01, *p* = 0.03) were each independently associated with an increased risk of maternal death. Among women who had any of these three criteria, mortality was 29.1% (25/86). There were no deaths in 88 pregnant women with severe malaria with hyperparasitaemia (> 10%) or severe anaemia (haematocrit < 20%) only. Excluding these 88 women without organ dysfunction resulted in a mortality of 20.8% (26/125). Sensitivity analyses showed similar results (Additional files 6 and 7: Table S4, S5).

**Table 3:** Univariable and multivariable penalised logistic regression on the potential prognostic factors for maternal death

		Univariable		Multivariable	
Characteristic	*N*	Odds ratio (95% CI)	*p*-value	Odds ratio (95% CI)	*p*-value
Age < 20 years	6/42	3.75 (0.95–14.90)	0.06		
20–29	3/83	Reference			
≥ 30	7/54	3.21 (0.85–12.08)	0.08		
Trimester First	2/22	0.94 (0.19–4.61)	0.94		
Second	7/71	Reference			
Third	8/73	1.13 (0.38–3.34)	0.82		
Postpartum	1/3	6.96 (0.76–63.66)	0.09		
Gravidity primigravida	4/46	Reference			
Multigravida	6/77	0.86 (0.24–3.03)	0.81		
Presence of gametocytes	4/40	3.69 (0.71–19.28)	0.12		
No	2/90	Reference			
Presence of schizont	1/17	1.93 (0.28–13.16)	0.50		
No	4/99	Reference			
Presence of pigment^a^	7/103	2.41 (0.12–48.57)	0.57		
No	0/29	Reference			
Fever > 37.5 °C	9/99	0.89 (0.29–2.76)	0.84		
No	7/84	Reference			
Coma	22/70	11.40 (3.56–36.50)	< 0.0001	7.18 (2.01–25.57)	0.002
No	4/141	Reference		Reference	
Severe anaemia	6/69	1.44 (0.47–4.47)	0.52		
No	14/132	Reference			
Renal failure	5/17	4.79 (1.21–18.99)	0.026		
No	13/171	Reference			
Respiratory failure	5/20	12.95 (3.16–53.12)	0.0004	4.98 (1.13–22.01)	0.03
No	16/171	Reference		Reference	
Convulsion	1/7	2.70 (0.38–19.31)	0.32		
No	21/192	Reference			
Metabolic acidosis^b^	7/35	2.10 (0.54–8.16)	0.28		
No	9/55	Reference			
Jaundice	5/30	1.37 (0.40–4.70)	0.62		
No	16/172	Reference			
Hypoglycaemia	3/12	3.66 (0.79–16.95)	0.10		
No	17/137	Reference			
Hypotension	2/6	10.83 (1.84–63.89)	0.009	11.21 (1.27–98.92)	0.03
No	18/192	Reference		Reference	
Hyperparasitaemia	8/86	1.40 (0.49–4.02)	0.53		
No	13/120	Reference			
Prostration	4/18	2.74 (0.39–19.19)	0.31		
No	4/98	Reference			

*CI* confidence interval

Penalised logistic regression with fixed-effects for each study site was used. Multiple imputation was used for multivariable models. Calendar year and treatment (quinine-based or artemisinin-based) were adjusted in multivariable models

^a^When only patients with information on malaria pigment quantitatively assessed, the unadjusted odds ratio of maternal deaths for women with pigment in neutrophil (> 5%) was 2.33 (3/22 vs 2/29, 95%CI 0.34–15.83, *p* = 0.39)

^b^Variables not assessed for multivariable model because missing > 50%

### Pregnancy outcomes in the SMRU cohort

**Fig. 2:**
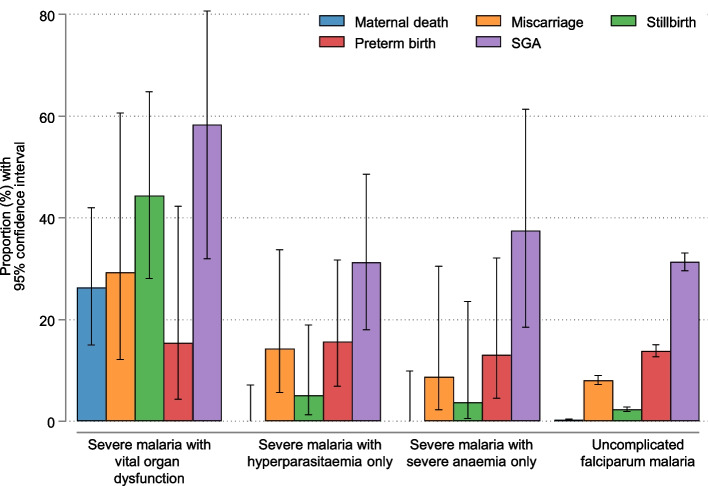
Outcomes of falciparum malaria in pregnancy in women attending the Shoklo Malaria Research Unit (SMRU) antenatal clinics on the Thailand-Myanmar border. SGA: small for gestational age

In the SMRU cohort, pregnancy outcomes were available for 82.9% (97/117) of women excluding three postpartum episodes, which were all live-born (Table 4). Foetal loss occurred in 23.3% (25/117, 95% CI 16.4–32.6% by Kaplan–Meier estimator) of pregnancies: 10 miscarriages and 15 stillbirths. The proportion of miscarriages among those women who had severe malaria before 28 weeks gestation was 15.7% (10/65, 95% CI 8.8–27.2%) and was highest in the severe malaria with organ dysfunction group (29.3%, 4/14, 95% CI 12.1–60.6%) (Table 4). Stillbirth occurred in 16.1% (15/99, 95% CI 10.0–25.3%) of pregnancies followed up beyond 28 weeks and was the highest in the severe malaria with organ dysfunction group (44.4%, 12/28, 95% CI 28.1–64.8%). By comparison in women attending the same antenatal clinics, the prevalence of miscarriage and stillbirth among women with uncomplicated falciparum malaria in pregnancy was 8.0% (296/3692) and 2.3% (106/4616) respectively (Fig. 2).

**Table 4:** Summary of the maternal and foetal outcomes at the Shoklo Malaria Research Unit (SMRU) on the Thailand-Myanmar border

Outcome	All	Severe malaria with vital organ dysfunction	Severe malaria with hyperparasitaemia only	Severe malaria with severe anaemia only
Delivery outcomes assessed	83% (97/117)	91% (30/33)	84% (41/49)	74% (26/35)
Foetal loss^a^	23% (25/117)	51% (16/33)	13% (6/49)	9% (3/35)
Miscarriage (< 28 weeks)^a^	16% (10/65)	29% (4/14)	14% (4/28)	9% (2/23)
Stillbirth (≥ 28 weeks)^a^	16% (15/99)	44% (12/28)	5% (2/42)	4% (1/29)
Day to foetal loss	-	1 [0–3]	0, 0, 1, 3, 3, 54	23, 30, 90
Live-born	72	14	35	23
Estimated gestational age at delivery	39.3 [38.1–40.2]	39.4 [38.0–39.5]	39.5 [38.1–40.1]	39.2 [37.5–40.4]
Preterm delivery (< 37 weeks)^b^	15% (10/68)	15% (2/13)	16% (5/32)	13% (3/23)
Sex (boy)	53% (38/72)	50% (7/14)	51% (18/35))	56% (13/23)
Congenital abnormality	1/72	0/14	1/35	0/23
Birthweight^c^	2700 [2345–3000] (*n* = 60)	2530 [2050–2970] (*n* = 12)	2745 [2510–3075] (*n* = 32)	2550 [2335–3000] (*n* = 16)
SGA^c^	38% (23/60)	58% (7/12)	31% (10/32)	38% (6/16)
Z-score^c^	− 1.1 [− 1.7 to − 0.57] (*n* = 60)	− 1.3 [− 2.1 to − 0.71] (*n* = 12)	− 0.91 [− 1.5 to − 0.21] (*n* = 32)	− 1.2 [− 1.9 to − 0.62] (*n* = 16)
LBW (< 2500 g)^c^	30% (18/60)	42% (5/12)	19% (6/32)	44% (7/16)

*LBW* low birth weight, *SGA* small-for-gestational-age

Median [interquartile range] or percentage (number of outcome / number evaluated) are presented. Postpartum episodes were excluded from this table. Only the last episodes were included from those who had multiple episodes. INTERBROWTH-21st international standard growth chart was used for SGA (defined as < 10%) and *Z*-score of birthweight for gestational age

^a^Estimated by Kaplan–Meier method. Miscarriage includes women who had severe malaria before 28 weeks, and stillbirth includes women who were followed up beyond 28 weeks

^b^Four women who had severe malaria on or after the 37th gestational week were excluded

^c^Only live-born singletons without congenital abnormality included. Birthweights weighed > 14 days after delivery were excluded

All 72 live-born babies were singletons, and there was one congenital abnormality (unilateral cleft lip and palate). Median estimated gestational age at delivery was 39.3 (IQR 38.1–40.2) weeks. Among women who had severe malaria before the 37th week of gestation and did not have foetal loss, the overall prevalence of preterm birth was 14.7% (10/68), which was not different from 13.8% (463/3353) in pregnant women who had uncomplicated falciparum malaria before the 37th week of gestation.

Median birthweight was 2700 g (IQR 2345–3000 g, *n* = 60). The proportion of SGA was 38.3% (23/60). Compared with those who had uncomplicated falciparum malaria in pregnancy (31.3%, 831/2652), the proportion of SGA was higher in the severe malaria with organ dysfunction group (58.3%, 7/12), but not different in the groups with hyperparasitaemia only (31.3%, 10/32) or severe anaemia only (37.5%, 6/16). These figures were similar when only those whose birthweight had been assessed within 3 days of birth were included (Additional file 8: Table S6).

### Timing of the adverse outcomes in the SMRU cohort

**Fig. 3:**
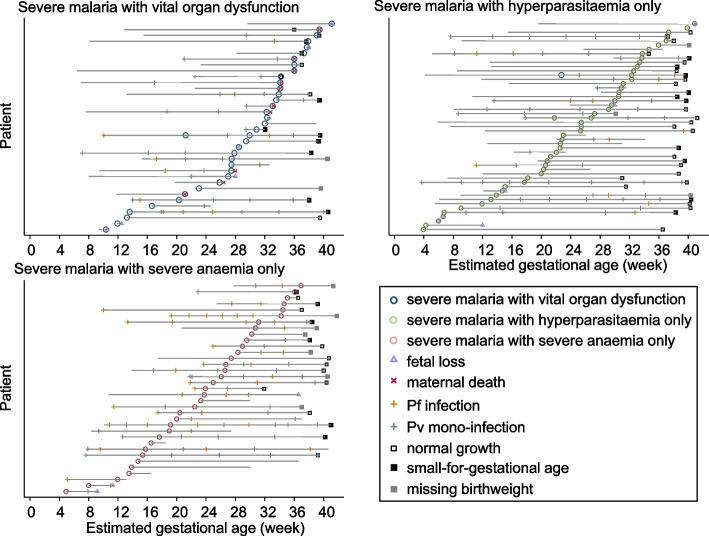
Gestational age timing of severe malaria, severe anaemia and hyperparasitaemia with identification of other malaria episodes, maternal death and foetal outcomes per pregnancy, at the Shoklo Malaria Research Unit (SMRU) on the Thailand-Myanmar border. Pf: *Plasmodium falciparum*. Pv: *Plasmodium vivax*

Foetal losses occurred in the first week of severe malaria illness in the majority (84.0%, 21/25) of cases (Fig. 3): all 16 in the severe malaria with organ dysfunction group; 5/6 in the severe malaria with hyperparasitaemia only group; and 0/3 in the severe malaria with severe anaemia only group. In the severe malaria with organ dysfunction group, 44.7% (17/38) had an adverse outcome in the week following diagnosis (nine maternal deaths, seven foetal losses without maternal deaths and one preterm birth). In the severe malaria with hyperparasitaemia only group, 12.0% (6/50) had an adverse outcome within a week (five with foetal loss and one preterm birth) whereas in the severe anaemia only group, only one (2.9%, 1/35) had an adverse outcome within a week: preterm birth at 36.3 gestational weeks.

## Discussion

Severe falciparum malaria is an important cause of maternal death in malaria-endemic areas, particularly in lower-transmission areas such as those in most of Asia [2, 11]. The overall maternal mortality in severe malaria in pregnancy in Asia including the current study was estimated to be 25.1% (19 cohorts, range 0–70.6%, 95% CI 15.2–36.3%, *I*^2^ 87.1%). This study included two different series of pregnant women with severe falciparum malaria, one community-based and the other hospital-based, showed clearly that, whereas falciparum malaria causing vital organ dysfunction carries a high mortality, severe anaemia or hyperparasitaemia only can present in ambulant women and carry a good prognosis if they are treated properly. Indeed, oral treatment under careful supervision was sufficient in 70% of the groups with either hyperparasitaemia only or severe anaemia only. This marked difference in mortality between the different WHO severe malaria criteria probably explains the variation in reported malaria-related mortality in pregnancy in the international literature, and it raises the question of whether these should be included in the criteria for a diagnosis of severe malaria in pregnancy.

Definitions of severe malaria are valuable in triage and in prompting emergency treatment. In this study, coma, respiratory failure and hypotension were each independently associated with mortality in pregnant women. Similar findings have been reported previously in both children and adults showing that coma, increased blood urea, metabolic acidosis, shock and pulmonary oedema were associated with increased mortality [40]. In areas of high transmission, severe anaemia is the most common reason for hospitalising young children with malaria [1, 41], who bear the brunt of global severe malaria mortality [2, 3]. Anaemia is also very common in pregnant women and is often multifactorial [41]. Pregnant women who are already anaemic may have a further reduction in haemoglobin when infected with malaria, and thereby meet the severe anaemia criterion for severe malaria. Malaria itself can cumulatively increase the risk of anaemia [41] despite routine ANC screening for anaemia and provision of haematinics for mild anaemia (haematocrit < 30%). In the current study, women presenting with severe anaemia alone commonly had multiple episodes of malaria before developing severe anaemia. In a study of 418 pregnant women with malaria at SMRU, only 2% did not have anaemia (haematocrit < 30%) detected between day 0 and day 42 [42]. Severe anaemia (haematocrit < 20% or haemoglobin < 7 g/dL) is often reported as the most common presenting sign of severe malaria in pregnant women [8, 10]: while this has adverse consequences for the foetus increasing the risk of foetal loss and foetal growth restriction, the anaemia is often well compensated and, if maintained in the 5–7 g/dL range, may not pose a risk for the mother—particularly when blood transfusion is available. Life-threatening anaemia (< Hb 4 g/dL) [7] was uncommon in this study (2.5%, 5/198). A reconsideration of haemoglobin thresholds, particularly in pregnancy, may be warranted.

Hyperparasitaemia reflects low immunity against falciparum malaria, such as in younger children or pregnant women [1, 3, 43]. Although higher parasitaemia is generally associated with severe clinical manifestations, the relationship is not linear [3, 41, 44]. Previous studies have shown that uncomplicated hyperparasitaemia can be treated successfully with oral ACTs without mortality [19, 45], although longer courses of artemisinin derivatives (5–7 days) [19] and close monitoring are required. The relationship between parasite density and mortality is complex and depends on background immunity, stage of parasite development at presentation and access to artemisinin-containing antimalarials, which prevent circulating young ring stage parasites maturing and resulting in red cell sequestration [2, 3]. The proportion of patients with severe malaria with hyperparasitaemia alone therefore affects the overall severe malaria mortality (particularly in the context of artemisinin use). The optimum cut-off point for the definition of hyperparasitaemia in pregnancy is unclear.

All three maternal deaths after parenteral artesunate/artemether in the SMRU cohort occurred within 24 h [4]. Patients who die rapidly may have presented too late in the disease process—emphasising the importance of providing parenteral artesunate promptly to suspected severe cases. In this cohort, the majority (61.8%) of severe episodes occurred in women who came to ANC for the first time or after more than 2 weeks of absence. Encouraging women to come to ANC early in the first trimester and improving regular attendance may reduce the incidence of severe malaria.

Increased risks of foetal loss and SGA after severe malaria in pregnancy, particularly among those with organ dysfunction, were reported previously in a small case series [30]. The majority of foetal losses (84.0%) occurred within the first week of diagnosis; 44.7% of the SMRU severe malaria with organ dysfunction group had either maternal death, foetal loss or preterm birth within 1 week. A higher risk of foetal loss and preterm birth in severe malaria compared with uncomplicated malaria has also been reported in sub-Saharan Africa [15].

There are several limitations to this study. First, some of the WHO severe malaria criteria were unavailable. Important determinants of outcome (e.g. renal failure and metabolic acidosis) could not be always assessed and blood glucose was not monitored frequently particularly when the patient was conscious. These missing criteria require laboratory investigation and are frequently unavailable at the primary healthcare level in malaria-endemic countries. The results of this study have practical utility for settings where most malaria is diagnosed and treated, i.e. in rural and remote settings. The number of fulfilled criteria [14] can be another useful indicator to be assessed if information is complete. Although co-morbidities that are known to be associated with maternal mortality were not measurable except for eclampsia/pre-eclampsia, HIV infection was an unlikely comorbidity on the Thailand-Myanmar border where the seroprevalence has been reported as < 0.5% [46]. Second, much of the analysis was based on a single cohort at SMRU where birth outcomes could be compared, whereas the hospital-based studies provided data only on maternal outcomes. At SMRU, early and regular screening and prompt effective treatment has been provided for both malaria and anaemia, which could have resulted in a lower incidence of severe malaria and lower mortality from severe anaemia compared with other studies. On the other hand, the substantial prevalence of vivax malaria in this area and the consequent frequent recurrences are significant contributors to anaemia in pregnancy [41]. Furthermore, the intensive care and specialist support, management and characteristics were different between two series. Pregnant women with hyperparasitaemia or severe anaemia only were systematically treated with oral antimalarials at SMRU consistent with treatment evidenced in non-pregnant patients in the same setting [19, 45], but even without intravenous treatment, these two groups had better prognosis than those with other severe malaria criteria. These differences reflect the heterogeneity and variability of reports of severe malaria in pregnancy in the past literature, which make comparisons with these previous studies difficult. Third, data of severe malaria in the postpartum period were limited in our study and also in the literature. Although the placenta, the site of sequestration of falciparum malaria parasites, no longer exists after delivery, hormonal changes take place over the several weeks of postpartum period, and little is known about the recovery of immunity which has been altered by pregnancy [47]. Finally, the cohorts included in this study were in Southeast Asian countries, where the transmission of malaria is low, as are levels of falciparum premunition in women of childbearing age. The observed lack of association between gravidity and maternal mortality among women who developed severe malaria might be explained by the failure to develop pregnancy-specific acquired immunity against parasites harbouring VAR2CSA, which causes placental malaria, in this low-transmission area [11, 43, 48]. This does, however, ensure that the probability of misdiagnosis of severe malaria (e.g. sepsis with coincidental parasitaemia) is less likely [3, 49]. The signs and symptoms associated with increased risks of maternal death by severe malaria we identified are also commonly seen in maternal sepsis [50]. The three risk criteria identified in this study coincide with those used in the quick SOFA score for screening for sepsis [51], highlighting the importance of these three signs in life-threatening infections. Identification of the causal pathogen is thus important. Although this pooled analysis included the largest numbers of pregnant women with severe malaria in Asia, the numbers were still small, increasing the beta error. Further studies will be needed to assess whether any other WHO severe criteria (e.g. renal failure) are independently associated with maternal mortality.

## Conclusions

Anaemia and malaria are common in pregnant women in tropical settings. Hyperparasitaemia is more common in low than high-transmission settings. However, anaemia and hyperparasitaemia often are not associated with concomitant vital organ dysfunction requiring parenteral malaria treatment. On the contrary, they carry a good prognosis for the mother with ready access to oral artemisinin treatments and if necessary, blood transfusion. The reported variability in the mortality of severe malaria in pregnancy is probably explained largely by the different prevalence of these two severe malaria criteria in different series. Maternal mortality is consistently high in severe malaria with vital organ dysfunction, notably cerebral malaria, requiring immediate life-saving intravenous artesunate administration. Severe malaria in pregnancy increases the immediate risk of foetal loss and preterm delivery, and the risk that the baby is born small-for-gestational-age.

Greater malaria control efforts in the non-pregnant population along the Thailand-Myanmar border has resulted in a significant decline in malaria in pregnancy and hence mortality in all patients from severe malaria, including pregnant women [17]. Waning immunity achieved through elimination efforts in this area makes pregnant women vulnerable to unpredictable programmatic break down due to war and pandemic. Intensive efforts to maintain access to ANC to minimise barriers to early diagnosis and treatment of malaria and anaemia are required.

### Supplementary Information

**Additional file 1:** **Table S1.** The World Health Organization Severe Malaria Criteria (from the current Guidelines for the treatment of malaria).

**Additional file 2:** **Table S2.** Treatment for each severe falciparum malaria sub-group group over time at the Shoklo Malaria Research Unit.

**Additional file 3.** Supplemental Methods of Multiple imputation.

**Additional file 4:** **Table S3.** The numbers and proportion of pregnant women who met each severe malaria criterion.

**Additional file 5:** **Figure S1.** Forest plot of the maternal mortality among pregnant women with severe malaria in Asia.

**Additional file 6:** **Table S4.** The adjusted odds ratio of maternal death by complete case analysis model and multiple imputation model.

**Additional file 7:** **Table S5.** Univariable and multivariable penalised logistic regression on the potential prognostic factors for maternal death using the WHO severe malaria criteria for research purposes.

**Additional file 8:** **Table S6.** Birthweight assessment by clinical severity groups on the Thailand-Myanmar border including only newborns who were weighed within three days from birth.

## Data Availability

Data will be available from the Mahidol-Oxford Tropical Medicine Research Unit Institutional data access committee upon reasonable request from researchers who meet the criteria for access to confidential data (https://www.tropmedres.ac/units/moru-bangkok/bioethics-engagement/data-sharing).

## Abbreviations

ANC: Antenatal care
CI: Confidence interval
EGA: Estimated gestational age
IQR: Interquartile range
LBW: Low birth weight
MORU: Mahidol-Oxford Tropical Medicine Research Unit
OR: Odds ratio
OUCRU: Oxford University Clinical Research Unit
RCT: Randomised controlled trial
SD: Standard deviation
SEAQUAMAT: Southeast Asian Quinine Artesunate Malaria trial
SGA: Small-for-gestational-age
SMRU: Shoklo Malaria Research Unit
WHO: World Health Organization
