# Supplementary material for: Severe falciparum malaria in pregnancy in Southeast Asia: a multi-centre retrospective cohort study
Source: BMC Med. 2023 Aug 24;21:320. doi: 10.1186/s12916-023-02991-8 (PMC10464355; doi:10.1186/s12916-023-02991-8)
Supplement: Supplementary file 1 — Additional file 1: Table S1. The World Health Organization Severe Malaria Criteria (from the current Guidelines for the treatment of malaria). [file 12916_2023_2991_MOESM1_ESM.pdf]

Table S1. The World Health Organization Severe Malaria Criteria (from current Guidelines for the treatment of malaria) [6]

For epidemiological purposes, severe falciparum malaria is defined as one or more of the following, occurring in the absence of an identified alternative cause and in the presence of *P. falciparum* asexual parasitaemia.

Criterion	WHO definition [6]	SMRU definition	SEAQUAMAT [4]	AQ [20]	AAV [21]
Impaired consciousness	A Glasgow coma score < 11 in adults or a Blantyre coma score < 3 in children	Coma or Glasgow coma score < 11 if assessed.	Same as WHO	Glasgow Coma Scale of less than 11	Glasgow Coma Scale of less than 11
Prostration†	Generalized weakness so that the person is unable to sit, stand or walk without assistance	Same as WHO	Not used as an enrolement criterion	Not used as an enrolement criterion	Not used as an enrolement criterion
Multiple convulsions†	More than two episodes within 24 h	Same as WHO	Not used as an enrolement criterion	Not used as an enrolement criterion	Not used as an enrolement criterion
Acidosis	A base deficit of > 8 mEq/L or, if not available, a plasma bicarbonate level of < 15 mmol/L or venous plasma lactate ≥ 5 mmol/L. Severe acidosis manifests clinically as respiratory distress (rapid, deep, laboured breathing)	Not available	Same as WHO (Blood bicarbonate <15 mmol/L)		Plasma lactate > 4 mmol/L or standard base excess > - 5 mmol/L, base deficit < 10 mmol/L
Hypoglycaemia	Blood or plasma glucose < 2.2 mmol/L (< 40 mg/dL)	Same as WHO	Same as WHO	Same as WHO	Same as WHO
Severe malarial anaemia	Haemoglobin concentration ≤ 5 g/dL or a haematocrit of ≤ 15% in children < 12 years of age (<	Same as WHO (Haemoglobin concentration ≤ 7 g/dL or a	Haematocrit < 20% with a parasite count of more than 100,000/µL*	Haematocrit < 20% with a parasite count of more than 100,000/µL*	Haematocrit < 20% with a parasite count of more than 100,000/µL*

	7 g/dL and < 20%, respectively, in adults) with a parasite count > 10 000/µL	haematocrit of ≤ 20% with a parasite count > 10 000/µL)			
Renal impairment	Plasma or serum creatinine > 265 µmol/L (3 mg/dL) or blood urea > 20 mmol/L	Anuria	Blood urea nitrogen >17 mmol/L	Urine output <400 mL per 24 hours; and serum creatinine, >3 mg/dL (250 µmol/L)	Oliguria and serum creatinine > 250 µmol/L
Jaundice	Plasma or serum bilirubin > 50 µmol/L (3 mg/dL) with a parasite count > 100 000/ µL	Jaudice by clinical assessment with a parasite count > 100 000/ µL	Visible jaundice and P falciparum parasitaemia >100 000/µL	Serum bilirubin, > 2.5 mg/dL (50 µmol/L), with a parasite count of more than 100,000/µL	Same as WHO
Pulmonary oedema	Radiologically confirmed or oxygen saturation < 92% on room air with a respiratory rate > 30/min, often with chest indrawing and crepitations on auscultation	Respiratory failure requiring supplemental Oxygen; or oxygen saturation < 92% on room air with a respiratory rate > 30/min	Respiratory distress (>32 breaths per min)	Not used as an enrolement criterion	Not used as an enrolement criterion
Significant bleeding	Including recurrent or prolonged bleeding from the nose, gums or venepuncture sites; haematemesis or melaena	Not systematically assessed	Not used as an enrolement criterion	Not used as an enrolement criterion	Not used as an enrolement criterion
Shock	Compensated shock is defined as capillary refill ≥ 3 s or temperature gradient on leg (mid to proximal limb), but no	Systolic blood pressure < 80 mm Hg	Shock, as assessed by admitting physician (low blood pressure and cool peripheries)	Systolic blood pressure < 80 mm Hg with cool extremities	Systolic blood pressure < 80 mm Hg with cool extremities

	hypotension. Decompensated shock is defined as systolic blood pressure < 70 mm Hg in children or < 80 mm Hg in adults, with evidence of impaired perfusion (cool peripheries or prolonged capillary refill)				
Hyperparasitaemia	*P. falciparum* parasitaemia > 10%	Same as WHO	Same as WHO	Same as WHO	Parasite count > 500,000/μL*

*In the current study, the same criterion as the WHO definition was used.

†Prostration and repeated convulsions are not included in the "research definition" of severe malaria in the WHO severe malaria supplement [2].
