# Supplementary material for: Severe falciparum malaria in pregnancy in Southeast Asia: a multi-centre retrospective cohort study
Source: BMC Med. 2023 Aug 24;21:320. doi: 10.1186/s12916-023-02991-8 (PMC10464355; doi:10.1186/s12916-023-02991-8)
Supplement: Supplementary file 2 — Additional file 2: Table S2. Treatment for each severe falciparum malaria sub-group group over time at the Shoklo Malaria Research Unit. [file 12916_2023_2991_MOESM2_ESM.pdf]

Table S2. Treatment for each severe falciparum malaria sub-group group over time at the Shoklo Malaria Research Unit

Start year	Treatment for pregnant women	
**First-line initial treatment for severe malaria with organ dysfunction**		
1989	Q IV: 20mg/kg (H0) over 4 hours followed by 10mg/kg over 2 hours every 8 hours	
1996	Q IV: 20mg/kg (H0) over 4 hours followed by 10mg/kg over 2 hours every 8 hours<br>Or<br>AM IM: 3.2 mg/kg (H0) followed by 1.6 mg/kg every 24 hours (or 8-12 hourly in 1996–7)	
2000	AS IV + (from 2006) clindamycin p.o.<br>H0: 2.4 mg, H12 and H24 1.2mg/kg and then 1.2 mg/kg every 24 hours<br>Or AM IM or Q IV if AS IV is unavailable	
2013	AS IV + clindamycin p.o.<br>2.4mg/kg at H0, H12, H24 and then every 24 hours<br>Suspected AS resistance or severe malaria with hyperparasitaemia (≥4% RBC)<br>Q IV + AS IV	
**First-line for severe malaria with hyperparasitaemia (>10% RBC) only (without other organ dysfunction)**		
1986	Q IV: 20mg/kg (H0) over 4 hours followed by 10mg/kg over 2 hours every 8 hours	
1994	AS p.o. 4mg/kg (D0), 2mg/kg (D1–2) and 1mg/kg (D3–6) (total dose 12 mg/kg)†	
1997	AS p.o. 4mg/kg (D0), 2mg/kg (D1–4) and 1mg/kg (D5–6) (total dose 16mg/kg) †	
1999	AS p.o. 4mg/kg (D0), 2mg/kg (D1–6) (total dose 16mg/kg) †	
2012	AS p.o. 4mg/kg (D0), 2mg/kg (D1–6) with DP p.o. 3 days (D4–6)*	
2013	AS p.o. 4 mg/kg (D0–2) followed by DP p.o. 5 days (D2–6)*	
2016	Any ACT extended regimen (for 7 days)*	
**First-line for severe malaria with severe anaemia only (without other organ dysfunction)**		
1989	All trimesters	Q7 or or MFQ p.o. (25mg/kg single dose)
1994	1st trimester<br>2nd & 3rd trimester	Q7<br>1st episode: Q7 or MFQ p.o. (25mg/kg single dose)<br>≥2nd episode: AS p.o. (2mg/kg D0–D4, 1mg/kg D5–6)
1996	1st trimester<br>2nd & 3rd trimester	Q7<br>AS p.o. (2mg/kg D0–D4, 1mg/kg D5–6) or MAS
2006	1st trimester<br>2nd & 3rd trimester	Q p.o. + clindamycin for 7 days<br>AS p.o. + clindamycin for 7 days
2017	All trimesters	Available ACT

AS: artesunate. AC: artesunate+clindamycin. D: day. DP: dihydroartemisinin+piperaquine. H: hour. IM: intramuscular. IV: intravenous. MAS:mefloquine+artesunate. MFQ: mefloquine. p.o.: per os. Q: quinine. RBC: red blood cells.
Clindamycin was given orally 5mg/kg three times a day for 7 days.
MAS: mefloquine 25 mg/kg in total + artesunate 4mg/kg for three days
Q7: Q p.o.: 10mg/kg three times a day for 7 days.
A rescue dose (1.2 mg/kg) was given if parasitaemia increased above the 95th percentile of the usual parasite clearance curve [19].
‡ Treatment was switched to oral drug once tolerated. Total 12–16 mg/kg (-2012) or 16–18 mg/kg (2013-) of AS or AM was given over 7 days.
† A partner drug (e.g. MFQ, or atovaquone-proguanil if available) was used whenever possible.
*First dose was given by IV or IM (2.4mg/kg) to those with asexual parasitaemia of >20% RBC.
