# Supplementary material for: Severe falciparum malaria in pregnancy in Southeast Asia: a multi-centre retrospective cohort study
Source: BMC Med. 2023 Aug 24;21:320. doi: 10.1186/s12916-023-02991-8 (PMC10464355; doi:10.1186/s12916-023-02991-8)
Supplement: Supplementary file 3 — Additional file 3. Supplemental Methods of Multiple imputation. [file 12916_2023_2991_MOESM3_ESM.pdf]

## Supplemental Methods of Multiple imputation

For missingness, joint-modelling multilevel multiple imputation was conducted using *jomo* command in R (R Foundation for Statistical Computing, Vienna, Austria) [24]. Missing variables were imputed for 50 times using random cluster-specific covariance matrices. All the variables listed in the Table 3, maternal death (the outcome), country, study, and treatment were included in the imputation model. Analysis results were pooled by Rubin's rules using *mi estimate* command in Stata MP 16.1 (Stata Corp, TX, US). Variables that were missing >50% were excluded from multivariable analyses as these variables may not be easily assessed in low resource settings.
