# Supplementary material for: Severe falciparum malaria in pregnancy in Southeast Asia: a multi-centre retrospective cohort study
Source: BMC Med. 2023 Aug 24;21:320. doi: 10.1186/s12916-023-02991-8 (PMC10464355; doi:10.1186/s12916-023-02991-8)
Supplement: Supplementary file 4 — Additional file 4: Table S3. The numbers and proportion of pregnant women who met each severe malaria criterion. [file 12916_2023_2991_MOESM4_ESM.pdf]

Table S3. The numbers and proportion of pregnant women who met each severe malaria criterion

WHO severe malaria criterion	SMRU cohort (n=123)	Hospital based cohorts (n=90)
Coma	19% (23/121)	52% (47/90)
Severe anaemia	43% (48/112)	24% (21/89)
Severe anaemia only	31% (35/112)	3% (3/89)
Renal failure	6% (6/100)	13% (11/88)
Respiratory failure	6% (7/109)	16% (13/82)
Convulsion	2% (2/109)	6% (5/90)
Severe metabolic acidosis	Not assessed	39% (35/90)
Jaundice	6% (7/112)	26% (23/90)
Hypoglycaemia	10% (6/61)	7% (6/88)
Hypotension	2% (2/110)	5% (4/88)
Hyperparasitaemia	53% (61/116)	26% (25/90)
Hyperparasitaemia only	41% (47/116)	1% (1/90)
Prostration	11% (12/110)	100% (6/6)
Severe bleeding	Not assessed	Not assessed

MORU: Mahidol-Oxford Research Unit, SMRU: Shoklo Malaria Research Unit, WHO: World Health Organization.

The denominator is the number of patients assessed or information available.

The numbers and proportions of missing information are as following: coma (2, 1%); severe anaemia (12, 6%); renal failure (25, 12%); respiratory failure (22, 10%); convulsion (14, 7%); metabolic acidosis (123, 58%); jaundice (11, 5%); hypoglycemia (64, 30%); hypotension (15, 7%); hyperparasitaemia (7, 3%); prostration (97, 46%).
