# Supplementary material for: Severe falciparum malaria in pregnancy in Southeast Asia: a multi-centre retrospective cohort study
Source: BMC Med. 2023 Aug 24;21:320. doi: 10.1186/s12916-023-02991-8 (PMC10464355; doi:10.1186/s12916-023-02991-8)
Supplement: Supplementary file 5 — Additional file 5: Figure S1. Forest plot of the maternal mortality among pregnant women with severe malaria in Asia. [file 12916_2023_2991_MOESM5_ESM.pdf]

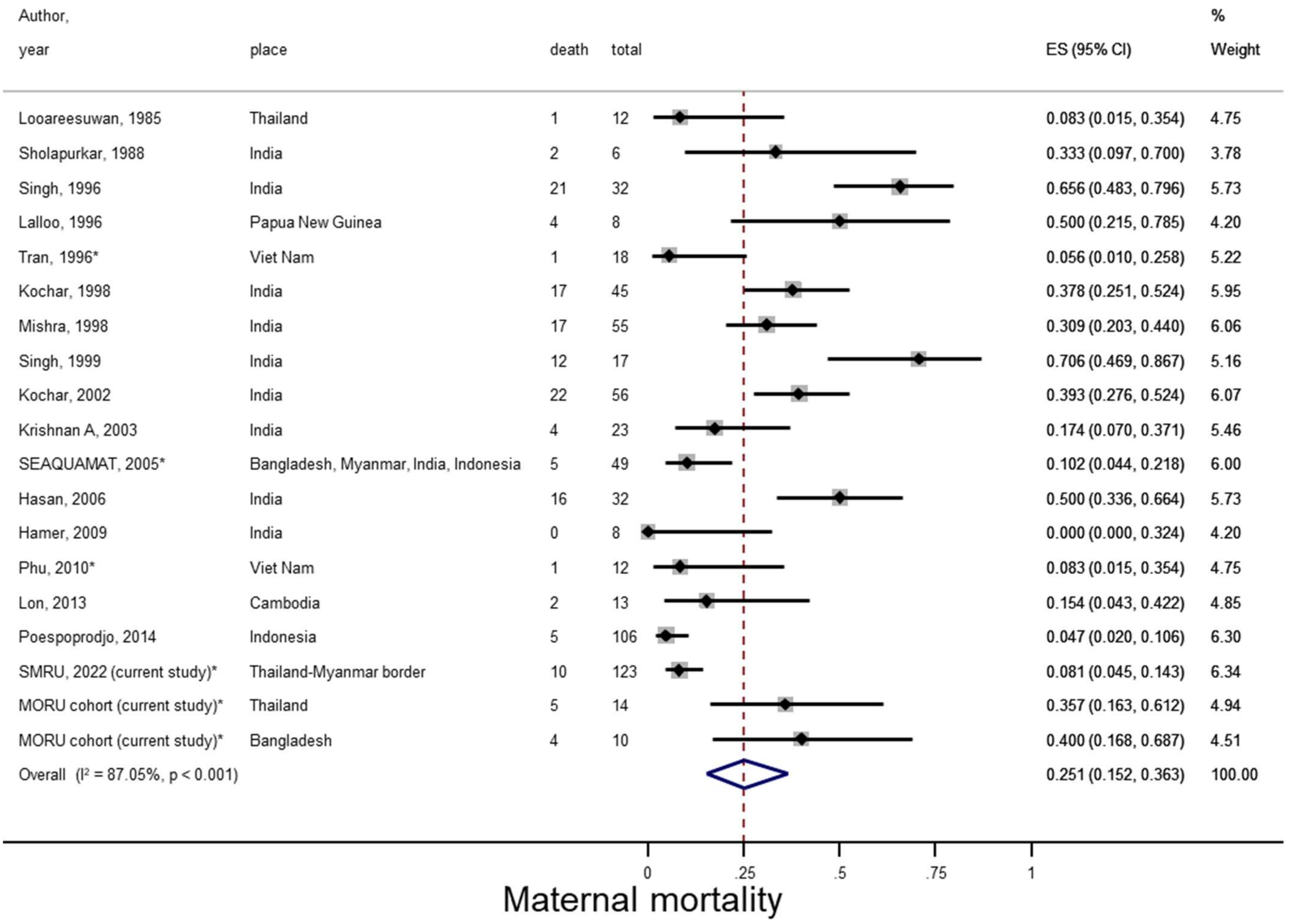

Figure S1. Forest plot of the maternal mortality among pregnant women with severe malaria in Asia

*included in the current study. MORU: Mahidol-Oxford Research Unit Tropical Health Network cohorts; SMRU: Shoklo Malaria Research Unit

Supplemental information on meta-analysis

In total, 19 studies (14 published studies [4, 10, 28-39] and five new cohorts in the current study) are included in the meta-analysis. In the current manuscript, one published study [4] reported mortality for pregnant women but two other studies [20, 21] did not. For the MORU cohorts included in the current study, women who did not strictly fulfill the WHO severe malaria definition (Table S1) are retained here. Four articles that reported the mortality among pregnant women with severe malaria were excluded from this meta-analysis because of multiple publications of the same cohort (Acta Leiden 1987, 55:115-120., Indian J Malariol 1995, 32(1):6-13.), or case reports (only three cases each) (J Assoc Physicians India 1989, 37(9):592-593., Int J Gynaecol Obstet 2007, 97(1):48-49.). In one study [31], the number of maternal mortality was reported but it was not clear whether they fullfilled severe malaria criteria. As the mortality from uncomplicated malaria is rare, we here assumed that all maternal mortality occurred in women with severe malaria in that study. Two studies were conducted at the same hospital in overlapping years, therefore there may be some cases counted twice [32, 38].
