# Supplementary material for: Severe falciparum malaria in pregnancy in Southeast Asia: a multi-centre retrospective cohort study
Source: BMC Med. 2023 Aug 24;21:320. doi: 10.1186/s12916-023-02991-8 (PMC10464355; doi:10.1186/s12916-023-02991-8)
Supplement: Supplementary file 6 — Additional file 6: Table S4. The adjusted odds ratio of maternal death by complete case analysis model and multiple imputation model. [file 12916_2023_2991_MOESM6_ESM.pdf]

Table S4. The adjusted odds ratio of maternal death by complete case analysis model and multiple imputation model

	Univariable	Multivariable	
		MI (n=213)	CC (n=187)
Coma	11.40 (3.56-36.50)	7.17 (2.01-25.57)	3.77 (0.96-14.82)
Respiratory failure	12.95 (3.16-53.12)	4.98 (1.13-22.01)	7.10 (1.49-33.74)
Hypotension	10.83 (1.84-63.89)	11.21 (1.27-98.92)	27.56 (2.46-308.33)

CC: complete case, MI: multiple imputation
