# Supplementary material for: Severe falciparum malaria in pregnancy in Southeast Asia: a multi-centre retrospective cohort study
Source: BMC Med. 2023 Aug 24;21:320. doi: 10.1186/s12916-023-02991-8 (PMC10464355; doi:10.1186/s12916-023-02991-8)
Supplement: Supplementary file 7 — Additional file 7: Table S5. Univariable and multivariable penalised logistic regression on the potential prognostic factors for maternal death using the WHO severe malaria criteria for research purposes. [file 12916_2023_2991_MOESM7_ESM.pdf]

Table S5. Univariable and multivariable penalised logistic regression on the potential prognostic factors for maternal death using the WHO severe malaria criteria for research purposes

		Univariable		Multivariable	
Characteristic	N	Odds ratio (95% CI)	p-value	Odds ratio (95% CI)	p-value
Age <20 years	6/39	3.97 (1.00-15.77)	0.05		
20-29	3/82	Reference			
≥30	7/53	3.23 (0.86-12.12)	0.08		
Trimester First	2/21	0.93 (0.18-4.70)	0.93		
Second	7/69	Reference			
Third	8/70	1.23 (0.41-3.66)	0.71		
Postpartum	1/3	6.90 (0.75-63.40)	0.09		
Gravidity primigravida	4/43	Reference			
Multigravida	6/75	0.82 (0.23-2.91)	0.76		
Presence of gametocytes	4/38	3.63 (0.69-19.01)	0.13		
No	2/86	Reference			
Presence of schizont	1/16	1.97 (0.29-13.49)	0.49		
No	4/95	Reference			
Presence of pigment†	7/97	2.60 (0.13-52.46)	0.53		
No	0/29	Reference			
Fever >37.5 C	9/93	0.98 (0.31-3.09)	0.98		
No	7/84	Reference			
Coma	22/70	10.72 (3.34-34.44)	0.0001	6.86 (1.91-24.66)	0.003
No	4/136	Reference		Reference	
Severe anaemia	6/69	1.34 (0.43-4.18)	0.61		
No	14/126	Reference			
Renal failure	5/17	4.60 (1.15-18.39)	0.03		
No	13/167	Reference			
Respiratory failure	5/20	12.60 (3.06-51.85)	0.0004	5.01 (1.15-21.90)	0.03
No	16/167	Reference		Reference	
Convulsion	1/7	2.68 (0.38-19.12)	0.32		
No	21/188	Reference			
Metabolic acidosis*	7/35	1.90 (0.48-7.49)	0.36		
No	9/54	Reference			
Jaundice	5/30	1.28 (0.37-4.43)	0.70		
No	16/168	Reference			

Hypoglycaemia		3/12	3.53 (0.77-16.27)	0.11		
	No	17/132	Reference			
Hypotension		2/6	10.67 (1.82-62.72)	0.009	11.44 (1.15-99.56)	0.03
	No	18/187	Reference		Reference	
Hyperparasitaemia		8/86	1.27 (0.44-3.70)	0.66		
	No	13/114	Reference			
Prostration		4/12	4.85 (0.65-36.22)	0.12		
	No	4/98	Reference			

CI: confidence interval. EGA: estimated gestational age.

Penalized logistic regression with fixed-effects for each study site is used. Multiple imputation is used for multivariable models. Calendar year and treatment (quinine-based or artemisinin-based) are adjusted in multivariable models.

* Variables not assessed for multivariable model because missing >50%.

† When only patients with information on malaria pigment quantitatively assessed, the odds ratio of maternal deaths for women with pigment in neutrophil (>5%) was 2.33 (3/22 vs 2/29, 95%CI 0.34-15.83, p=0.39).

Sensitivity analyses excluding women with convulsions or prostration

WHO has two criteria for severe malaria. The one used in the manuscript is for epidemiology and clinical management, defined in the WHO (treatment) guidelines [6]. The other one was for research purposes, defined in 2014 [2]. The difference between them is that two criteria, multiple convulsions and prostration, are not included in the latter.

Using the WHO 2014 severe malaria criteria for research purposes [2], the overall maternal mortality was 12.6% (26/207). Six pregnant women were excluded because they had prostration only. There were no maternal deaths among the excluded six women. The results of this sensitivity analysis are similar to the main results (Table S5).
