# Supplementary material for: Severe falciparum malaria in pregnancy in Southeast Asia: a multi-centre retrospective cohort study
Source: BMC Med. 2023 Aug 24;21:320. doi: 10.1186/s12916-023-02991-8 (PMC10464355; doi:10.1186/s12916-023-02991-8)
Supplement: Supplementary file 8 — Additional file 8: Table S6. Birthweight assessment by clinical severity groups on the Thailand-Myanmar border including only newborns who were weighed within three days from birth. [file 12916_2023_2991_MOESM8_ESM.pdf]

Table S6. Birthweight assessment by clinical severity groups on the Thailand-Myanmar border including only newborns who were weighed withing three days from birth

	All (n=42)	Severe malaria with vital organ dysfunction (n=9)	Severe malaria with hyperparasitaemia only (n=23)	Severe malaria with severe anaemia only (n=10)
Birthweight	2710 [2420-3000]	2300 [2000-2600]	2740 [2600-3050]	2900 [2340-3100]
SGA	43% (18/42)	67% (6/9)	39% (9/23)	30% (3/10)
Z-score	-1.2 [-1.8 - -0.69]	-1.4 [-2.4 - -1.2]	-1.1 [-1.6 - -0.69]	-0.88 [-1.8 - -0.59]
LBW (<2500g)	26% (11/42)	56% (5/9)	9% (2/23)	40% (4/10)

Median [interquartile range] or percentage (number of outcome / number evaluated) are presented.
LBW: low birth weight. SGA: Small for gestational age, SMRU: Shoklo Malaria Research Unit.INTERBROWTH-21st international standard growth chart was used for SGA (defined as <10%) and Z-score of birthweight for gestational age. Only live born singletons without congenital abnormality were included.
